# Age as an Independent Risk Factor for Diabetic Peripheral Neuropathy in Chinese Patients with Type 2 Diabetes

**DOI:** 10.14336/AD.2018.0618

**Published:** 2019-06-01

**Authors:** Fei Mao, Xiaoming Zhu, Siying Liu, Xiaona Qiao, Hangping Zheng, Bin Lu, Yiming Li

**Affiliations:** ^1^Department of Endocrinology and Metabolism, Huashan Hospital, Fudan University, Shanghai, China; ^2^Department of Endocrinology and Metabolism, Jing’an District Center Hospital of Shanghai, China

**Keywords:** Age, T2DM, DPN, risk factor

## Abstract

Type 2 diabetes mellitus (T2DM) is more prevalent in aging populations. Older adults with diabetes have higher rates of macro and micro vascular complications. Our study assessed whether age is an independent factor for both large and small nerve dysfunctions in Chinese patients with T2DM. This cross-sectional study involved a total of 950 patients with type 2 diabetes (mean age: 60.01±12.30 years). Diabetic peripheral neuropathy (DPN) was assessed according to clinical symptoms and physical examinations by using neuropathy symptom score (NSS), the neuropathy disability score (NDS), Michigan Neuropathy Screening Instrument (MNSI score), vibration perception threshold (VPT) and SUDOSCAN test. By using independent logistic regression model, we showed that age was an independent risk factor of DPN (odds ratio [OR] = 1.036, 95% confidence interval [CI] 1.018-1.054, P< 0.01). T2DM patients over 71 years had a higher risk of DPN determined by using NSS/NDS (OR= 2.087; 95% CI 1.112-3.918; P <0.05), MNSI (OR=1.922; 95% CI 1.136-3.252; P<0.05), VPT (OR=3.452; 95%CI 1.052-11.332; P<0.05) and SUDOSCAN (OR=1.922; 95%CI 1.136-3.252; P<0.05) as diagnostic criteria respectively. The results of spline analysis showed a non-linearly positive association between age and OR of DPN. Individuals with 40, 50, 60, and 70 years old had LnOR of 1.22 (95%CI: 0.44- 2.00), 1.79(95%CI: 0.67- 2.91), 2.29 (95% CI: 0.98- 3.59), and 2.67(95% CI: 1.38-3.96) in DPN risk compared to T2DM patients with 19 years old, respectively. All of the above results in our study suggested age as an independent risk factor for the development of diabetic neuropathy in T2DM patients is significantly associated with the occurrence of both small and large nerve dysfunction, independent of other risk factors.

Diabetes mellitus (DM) is a common metabolic disorder closely associated with chronic macro-micro vascular complications (WHO 2016). Among a series of chronic microvascular complications, diabetic peripheral neuropathy (DPN) is very common [1-3]. The typical DPN is a chronic, symmetrical, length-dependent sensorimotor polyneuropathy [3]. Up to 50% of patients experience typical manifestations consist of a series of sensory symptoms, which could be assessed by several traditional questionnaires as easy screening methods in everyday clinic [4-6].

Though the mechanism of DPN still remains unclear, it has already been proposed that inflammation, oxidative stress, and mitochondrial dysfunction are three main alterations involved in the pathologic changes of DPN [7]. All of these alterations are related to the process of aging [8]. DPN has been testified to be associated with a number of modifiable and non-modifiable risk factors [9-12]. Age as the most evaluated non-modifiable risk factor in the majority of epidemiological studies of DPN, has been found highly correlated with the incidence of DPN in T2DM patients [8].

Previous studies mainly used traditional screening methods as well as clinical golden diagnostic method NCS as criteria in diagnosing DPN [13]. However, an accurate assessment of small fiber damage in diabetic patients has not evolved in parallel with that of large fiber damage[13]. The aim of our study was to assess whether age is an independent factor for the occurrence of DPN including both small and large nerve dysfunction to prove more sufficient evidence for early screening of DPN in diabetic patients in elder patients.

## MATERIALS AND METHODS

### Study population

The study was conducted in Huashan hospital, Shanghai from September 2014 to September 2015. The ethics committee of Hua Shan Hospital approved the study. Voluntary outpatients diagnosed with type 2 diabetes between 18 and 80 years of age, with or without symptoms of neuropathy, were continually enrolled in the study. Exclusion criteria included undiagnosed hyperglycemia, T1DM patients, those under treatment with drugs that could have an effect on the sympathetic system such as beta blockers and antineoplastic drugs, implantation of electrical implantable devices, history of seizures or epilepsy, lumbar sciatic nerve lesion, severe varices of the lower limbs, other metabolic diseases including thyroid disease or vitamin B12 deficiency, and any other advanced systemic condition including severe hepatic and renal dysfunction [14, 15].

### Physical examination

One trained nurse examined all the patients and recorded the results. Basic physical characteristics were recorded including height, weight, waist and hip circumference measured by using standard methods. Body mass index (BMI) and waist hip ratio (WHR) were calculated. Blood pressure was recorded in the supine position after 5 minutes of rest. Medical history (diabetes, hypertension, dyslipidemia, cardiovascular disease and other) was recorded completely for each patient.

### Laboratory examination

Blood samples were collected after at least 8 hours of fasting. Plasma HbA1c level was determined by high-pressure liquid chromatography and liquid enzymatic assay. Serum total cholesterol (TC), triglyceride, high-density lipoprotein (HDL-C) cholesterol, triglycerides (TG), high-density lipoprotein cholesterol (HDL-C), low-density lipoprotein cholesterol (LDL-C) was measured by using an automatic analyzer (AU640; Olympus Corporation, Tokyo, Japan).

### Peripheral neuropathy examination

Symptoms and signs of lower limbs were recorded respectively. The assessments of the DPN were performed by one expert nurse using three different questionnaires including neuropathy symptom score (NSS), the neuropathy disability score (NDS) and Michigan Neuropathy Screening Instrument (MNSI score). A composite score was calculated separately for neuropathic symptoms using NSS score questionnaire and for clinical examination using NDS score. Neurological symptoms and signs based on the neuropathy symptom score (NSS) and the neuropathy disability scores (NDS) were evaluated. Neurological symptoms included burning, numbness, tingling, fatigue, cramping or aching, and neurological signs included vibration sense, pain, temperature sensation and ankle reflex.

MNSI score consists of two parts: The appearance of the feet (deformity, dry skin, callus, infection or fissures) and examination of foot ulceration, ankle reflex and vibration perception with a 128 Hz tuning fork. Evaluation of each parameter was made at both sides with a maximum score of 8 points.

### Vibration perception threshold (VPT) test

Vibration perception threshold was measured by the same technician by using a neuro-thesiometer (Bio-Thesiometer; Bio-Medical Instrument Co., Newbury Ohio). Before testing, skin temperature of each patient was examined by a nurse. Then, the stimulus of neuro-thesiometer was applied to the great toe with the probe balanced vertically on the pulp of the toe on each side. Patients were requested to indicate when vibration sensation was first perceived. Stimulus strength was gradually increased from null intensity to a value in voltage at which the subject first detected vibration. The whole testing procedure was carried out with the subject’s eyes closed. Both feet were tested three times in a random order and the VPT for each foot was determined as the average value of the three measurements calculated in volts. A ‘null stimulus’ trial was added before the testing to ensure the subject’s adherence and understanding. The whole testing generally required less than 3 min.

**Table 1:** Baseline characteristics of 950 patients of T2DM enrolled in the study.

Clinical characteristics	Mean ± SD
Age (years)	60.01±12.30
Male/ Female	555/395
Duration of T2DM (years)	8.85±7.33
SBP (mmHg)	128.69±13.63
DBP (mmHg)	80.27±7.89
HbA1c (%)	8.02±1.88
BMI (kg/m^2^)	24.43±3.59
Waist circumference (cm)	89.92±10.71
Hip circumference (cm)	96.61±6.62
WHR	0.93±0.07
Smokers (N, %)	215 (22.5)
Alcoholic (N, %)	126 (13.3%)
CHO (mmol/L)	4.48±1.23
HDL-C (mmol/L)	1.06±0.37
TG (mmol/L)	1.91±1.93
LDL-C (mmol/L)	2.54±0.88
NSS score	2.86±2.71
NDS score	2.85±2.62
VPT(V)	12.07±8.03
Foot ESC (µS)	62.25±19.28
Hand ESC (µS)	62.45±17.58

Data are means (SD), percentage (%);

T2DM, type 2 diabetes mellitus; BMI, body mass index; SBP, systolic blood pressure; DBP, dilated blood pressure; DPN, diabetic peripheral neuropathy; WHR, waist-hip ratio; HbA1c, glycated hemoglobin; LDL-C, low-density lipoprotein cholesterol; HDL-C, high-density lipoprotein cholesterol; CHO, cholesterol; TG, triglyceride; NSS, neuropathy symptom score; NDS, neuropathy disability score; MNSI, Michigan Neuropathy Screening Instrument; VPT, vibration perception threshold; ESC, electrochemical skin conductance; P-value was calculated after adjustment for age, sex except for itself. *P<0.05; ** P <0.01.

### SUDOSCAN test procedure

The SUDOSCAN device is composed of two sets of electrodes for the feet and hands, both of which are connected to a computer for recording and data analysis. The whole process of the test is non-invasive, and no special preparation is required. Patients only need to place the palms of their hands and the soles of their feet on the electrodes for 2 to 3 minutes and a low-voltage (<4V) electrical current stimulus will be applied by the device automatically [16]. The device can measure electrochemical skin conductance values expressed in micro-Siemens (μS) for the hands and the feet (both right and left sides). We used the mean of left and right ESC values for statistical analysis [16].

### Diagnostic criteria of DPN

The diagnostic criterion of DPN was a MNSI examination score of > 2 as previously reported [17]. In this study, we also used other diagnostic criteria of DPN including NSS/NDS score [18], VPT [19] and SUDOSCAN [16].

NSS/NDS scores: Patient with an NSS of 3-5 points were considered with mild neuropathy signs, 6-8 points as medium neuropathy signs and 9-10 points as severe neuropathy signs. DPN was diagnosed with an NDS score of ≥ 6, or an NDS score of 3-5 associated with an NSS score of ≥ 5. For VPT, we used a threshold of 15V as the cut-off for diagnosis of DPN in this study. As for the diagnostic criteria of SUDOSCAN test, we used 60 μS of mean feet ESC as the cut-off for diagnosis of DPN according to previous studies.

### Statistical analysis

Data are presented as means ± SD for normally distributed variables and as median (interquartile range) for variables with a skewed distribution. Parity and gender, which were analyzed by Chi-square distribution in Table 2. Differences between groups were examined by independent-sample t tests for normally distributed variables and Mann-Whitney U-test for non-normally distributed data. Differences in offspring parity and sex distribution were examined by Chi-square test. Logistic regressions were used to estimate the odds ratio of DPN in each age group using the lowest group as the reference category adjusting for other covariates. Considering that the association between age and DPN risk might be nonlinear, restricted cubic spline (RCS) analysis was used to describe nonlinear relationships between the continuous age and the DPN risk. The RCS analysis uses piecewise cubic polynomials that are connected across different intervals of a continuous variable. We chose 3 knots at quantiles 0.050, 0.500 and 0.95. In RCS analysis, the least value of age was used as the referent, and the ORs of all other age versus the referent value were calculated and plotted against their respective age.

## RESULTS

In this study, a total number of 950 patients with type 2 diabetes were enrolled continuously (including 563 males and 387 females). Amongst these 950 patients with type 2 diabetes (mean age: 60.01±12.30 years, mean duration of type 2 diabetes: 8.85±7.33 years, mean HbA1C% level: 8.02±1.88 %). Clinical and biochemical characteristics of the 950 patients are described in Table 1. We divided the patients into DPN and non-DPN groups by using MNSI score as diagnostic criteria as indicated before (Table 2). T2DM patients in the study with DPN diagnosed by MNSI are older (P< 0.01), have longer duration of T2DM (P< 0.001), higher systolic blood pressure (P<0.001), higher NSS score (P<0.001), higher NDS score (P<0.001) and MNSI score (P<0.001).

**Table 2:** Characteristics of 950 T2DM patients enrolled in the study divided by DPN diagnosed by MNSI score.

Clinical characteristics	With DPN (N=264)	Without DPN (N=686)	P
Age (years)	63.78±10.91	57.73±12.47	0.000**

Male/Female	157/107	398/288	0.069
Duration of T2DM (years)	11.10±8.23	7.81±6.62	0.000**
SB P(mmHg)	131.82±15.33	127.81±13.10	0.000**
DBP (mmHg)	80.52±7.61	80.20±8.08	0.619
BMI (kg/m^2^)	24.46±3.39	24.36±3.63	0.741
Waist circumference (cm)	90.74±9.95	89.71±10.94	0.238
Hip circumference (cm)	96.91±6.89	96.50±7.85	0.517
HbA1c (%)	8.19±1.81	8.05±1.97	0.400
Smokers (%)	56(56/200)	147(147/573)	0.515
Alcoholic (%)	23(23/200)	93(93/573)	0.134
MNSI score	5.54±2.11	1.93±1.59	0.000**
NSS score	4.05±2.76	2.50±2.58	0.000**
NDS score	5.59±2.17	1.92±2.04	0.000**
VPT (V)	17.11±10.66	10.39±6.13	0.000**
Foot ESC (µS)	53.49±22.69	65.52±17.14	0.000**
Hand ESC (µS)	55.86±19.21	64.76±16.67	0.000**

Data are means (SD), percentage (%);

T2DM, type 2 diabetes mellitus; BMI, body mass index; SBP, systolic blood pressure; DBP, dilated blood pressure; DPN, diabetic peripheral neuropathy; WHR, waist-hip ratio; HbA1c, glycated hemoglobin; LDL-C, low-density lipoprotein cholesterol; HDL-C, high-density lipoprotein cholesterol; CHO, cholesterol; TG, triglyceride; NSS, neuropathy symptom score; NDS, neuropathy disability score; MNSI, Michigan Neuropathy Screening Instrument; VPT, vibration perception threshold; ESC, electrochemical skin conductance; P-value was calculated after adjustment for age, sex except for itself.

* P<0.05;

** P <0.01;

**Table 3:** Characteristics of 950 T2DM patients enrolled in the study stratified by four age groups (≤50, 51-60, 61-70, ≥71).

	≤50 (N=183)	51-60 (N=269)	61-70 (N=308)	≥71 (N=180)	P

Male (%)	128 (69.95%)	167 (62.08%)	167 (54.22%)	93 (51.67%)	0.001**
Duration of T2DM (years)	4.73±5.11	7.57±5.42	10.28±7.85	12.89±8.32	0.000**
SBP (mmHg)	125.09±11.77	126.46±14.09	130.62±13.46	132.42±13.47	0.000**
DBP (mmHg)	81.03±7.92	80.88±7.97	80.26±7.80	78.34±7.62	0.004**
BMI	25.77±4.11	23.84±3.32	24.39±3.36	24.05±3.48	0.000**
WHR	0.93±0.08	0.93±0.06	0.92±0.07	0.93±0.09	0.952
NSS	2.24±2.59	2.82±2.80	3.13±2.69	3.11±2.61	0.003**
NDS	2.15±2.37	2.62±2.53	3.16±2.67	3.64±2.68	0.000**
MNSI	1.14±1.30	1.39±1.27	1.86±1.45	2.16±1.48	0.000**
VPT (V)	8.34±6.78	11.11±7.50	13.47±7.97	17.18±7.91	0.000**
Foot ESC (µS)	65.00±19.85	65.32±17.78	62.11±18.67	55.35±20.12	0.000**
Hand ESC (µS)	64.91±17.89	63.47±16.04	62.45±17.82	58.29±18.19	0.002**
HbA1c (%)	8.39±2.11	8.031±1.90	7.98±1.85	7.67±1.61	0.009**

T2DM, type 2 diabetes mellitus; BMI, body mass index; SBP, systolic blood pressure; DBP, dilated blood pressure; DPN, diabetic peripheral neuropathy; WHR, waist-hip ratio; HbA1c, glycated hemoglobin; NSS, neuropathy symptom score; NDS, neuropathy disability score; MNSI, Michigan Neuropathy Screening Instrument; VPT, vibration perception threshold; ESC, electrochemical skin conductance;

P-value was calculated after adjustment for age, sex except for itself.

* P<0.05;

** P <0.01.

To understand the influence of age on the incidence of DPN, we further analyzed all clinical characteristics in T2DM patients stratified by age of ≤50, 51-60, 61-70, ≥ 71 (Table 3). As we could see from the table, T2DM aging above 71 have the longest duration of T2DM (P< 0.01), highest systolic blood pressure level (P<0.01) and highest scores including NSS (P< 0.01), NDS (P<0.01), highest VPT level (P< 0.01), lowest hands (P<0.01)and feet ESC levels (P<0.01).

We used independent logistic regression model to quantify significant risk factors for DPN. Multivariate logistic regression analysis showed that clinical factors including age (odds ratio [OR] = 1.036, 95% confidence interval [CI] 1.018-1.054, P< 0.01), duration of T2DM (OR= 1.034, 95% CI 1.007-1.062, P< 0.01), HbA1c level (OR= 1.121, 95% CI 1.018-1.054, P< 0.01) and systolic blood pressure (OR= 1.017, 95% CI 1.003-1.032, P< 0.05) were independent risk factors of DPN (Table 4).

After adjusting for duration of T2DM, gender, smoking, drinking, SBP, BMI, HbA1c, as compared with T2DM patients aged under 50 years old (reference), T2DM patients aged over 71 years had a higher risk of DPN determined by using NSS/NDS (OR= 2.087; 95% CI 1.112-3.918; P < 0.05), MNSI (OR=1.922; 95% CI 1.136-3.252; P <0.05), VPT (OR=3.452; 95% CI 1.052-11.332; P<0.05) and SUDOSCAN (OR=1.922; 95% CI 1.136-3.252; P<0.05) as diagnostic criteria respectively (Table 5). However, both T2DM patients aged between 50 and 60 years old as well as aged between 60 and 70 didn’t show significant higher risk of DPN by different diagnostic criteria (Table 5).

**Figure 1.**
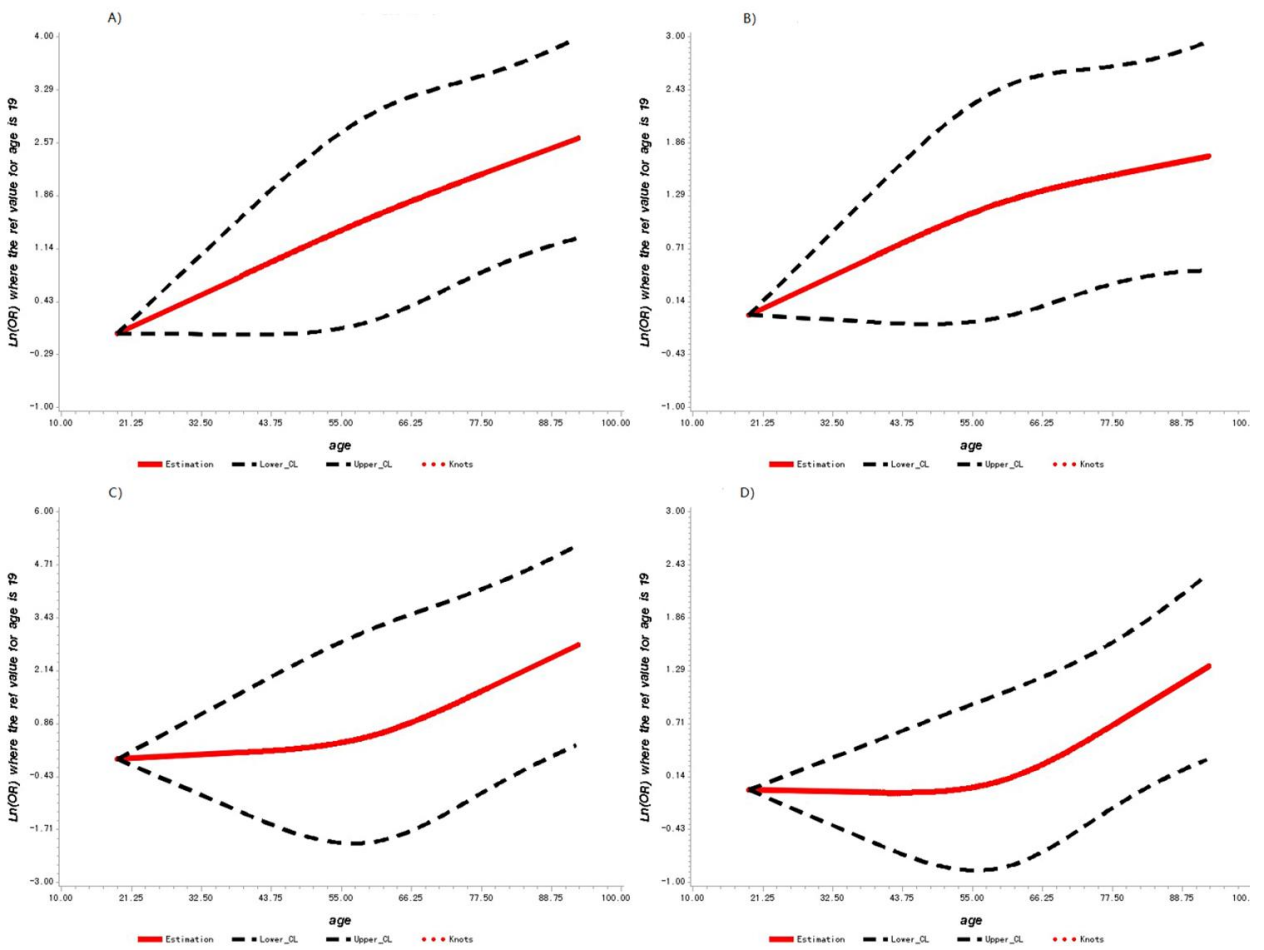
Adjusted dose-response association between age (years) and DPN diagnosed by different criteria including NSS/NDS score, MNSI score, VPT and SUDOSCAN Adjusted dose-response association between age (years) and the presence of DPN with three knots located at the 5^th^, 50^th^, and 95^th^ percentiles. Y-axis represents the Ln (Odds Ratio) to present DPN for any value of age compared to individuals with 19 years old. The red line is the adjusted curve and dashed lines are 95 percent confidence intervals. **A**) Adjusted dose-response association between age (years) and DPN diagnosed by MNSI score. **B**) Adjusted dose-response association between age (years) and DPN diagnosed by NSS/NDS score. **C**) Adjusted dose-response association between age (years) and DPN diagnosed by VPT value. **D**) Adjusted dose-response association between age (years) and DPN diagnosed by SUDOSCAN ESC value

We next used spline analysis to determine the risk association between age and OR of DPN in patients with T2DM. On spline analysis, the age level was non-linearly associated with OR of DPN comparing patients of 19 years old, and OR of DPN significantly increased with age (As seen in Figure 1). Individuals with 40, 50, 60, and 70 years old had LnOR of 1.22 (95% CI: 0.44, 2.00), 1.79 (95% CI: 0.67, 2.91), 2.29 (95% CI: 0.98, 3.59), and 2.67 (95% CI: 1.38-3.96) in DPN risk compared to T2DM patients with 19 years old, respectively.

## DISCUSSION

Diabetic neuropathy is a well-known microvascular complication of type 2 diabetes mellitus attributed to chronic hyperglycemia and is defined as the presence of peripheral nerve dysfunction in diabetics after exclusion of other causes [20, 21]. No study has ever reported the possible relationship between age and small fibre neuropathy which is indicated in early stage of diabetic nerve dysfunction. Our results in this study demonstrated that increasing age is independently associated with an increased risk of developing DPN in patients with T2DM by using different diagnostic criteria including both traditional scoring methods, VPT screening as well as the newly developed sudomotor device. We found that age is non-linearly positively associated with OR of DPN. This study is the first cross-sectional study carried out to analyze the risk factor of age in Chinese T2DM patients by using SUDOSCAN targeting at small nerve function and VPT targeting at large nerve function.

**Table 4:** Multivariate logistic regression model of clinical factors and DPN diagnosed by MNSI score.

Clinical factors	B	SE	OR	LCI	UCI	P
Age (years)	0.035	0.009	1.036	1.018	1.054	0.000**
Duration of T2DM (years)	0.034	0.014	1.034	1.007	1.062	0.015*
Gender	-0.149	0.198	0.862	0.585	1.270	0.452
SBP(mmHg)	0.017	0.007	1.017	1.003	1.032	0.015*
BMI (kg/m^2^)	0.014	0.028	1.014	0.960	1.071	0.618
HbA1c (%)	0.114	0.051	1.121	1.014	1.240	0.026*

T2DM, type 2 diabetes mellitus; BMI, body mass index; SBP, systolic blood pressure; DPN, diabetic peripheral neuropathy; HbA1c, glycated hemoglobin; SE, standard error; OR, odds ratio; CI, confidence interval; UCI, upper confidence interval; LCI, lower confidence interval

P-value was calculated after adjustment for age, sex except for itself.

* P<0.05;

** P <0.01.

Previous studies have already showed that sensory neuropathy is more common in long-standing diabetic subjects and is strongly related to age at diagnosis [10, 11, 22]. In year 2014, Anil Bhansali et al. [9] showed that age (OR 1.02, 95% CI 1.01-1.03, P < 0.001) was significantly associated with diabetic microvascular complications including diabetic neuropathy. Dehong Cai et al. [11]also showed that the prevalence of DPN in patients with age of 20-34, 35-49, 50-64 and ≥65 was 8.4%, 22.7%, 33.0% and 42.4%, respectively, which indicated a significant difference between age and the incidence of neuropathy (p<0.001). The study confirmed that age (OR: 1.016, 95%CI: 1.008, 1.024) was significantly associated with the development of DPN. Romulus Timar et al. [22] and their team showed in their study that the prevalence of DPN according to MNSI score was 28.8%, being significantly and positively correlated with higher age (65 vs 59 years; P=0.001) indicating that age influences the presence of DN, independent of other risk factors. As has been reported in many different animal studies, peripheral neuropathy which is detected by nerve conduction velocity, has been proved to be aging-related[23]. As nerve conduction velocity remains unchanged during adulthood of mice, it begins to decline after that. Further morphologic examination shows that there is a gradual decline in the number and density of both myelinated and unmyelinated nerve fibers in mice starting from 12-20 months old. And from 20 months on, there is approximately 50% loss of myelinated fibers and 35% loss of unmyelinated fibers in mice [24].

**Table 5:** Adjusted odds ratio of T2DM patients with DPN stratified by four age groups (≤50, 51-60, 61-70, ≥71) by using different diagnostic methods.

Age	NSS/NDS	P value	MNSI	P value	VPT	P value	SUDOSCAN	P value
≤50		Ref		Ref		Ref		Ref
51-60	1.740 (1.011-2.996)	0.046^*^	1.270 (0.810-1.992)	0.298	1.371 (0.430-4.376)	0.594	1.270 (0.810-1.992)	0.298
61-70	1.536 (0.880-2.681)	0.131	1.270 (0.804-2.005)	0.305	1.598 (0.505-5.060)	0.426	1.270 (0.780-2.218)	0.305
≥71	2.087 (1.112-3.918)	0.022^*^	1.922 (1.136-3.252)	0.015^*^	3.452 (1.052-11.332)	0.041^*^	1.922 (1.136-3.252)	0.015^*^

T2DM, type 2 diabetes mellitus; DPN, diabetic peripheral neuropathy; NSS, neuropathy symptom score; NDS, neuropathy disability score; MNSI, Michigan Neuropathy Screening Instrument; VPT, vibration perception threshold; SE, standard error; OR, odds ratio; CI, confidence interval; UCI, upper confidence interval; LCI, lower confidence interval;

P for One-way ANOVA. P-value was calculated after adjustment for duration of T2DM, gender, SBP, BMI, HbA1c.

*P<0.05.

The molecular basis of aging and aging-related changes is still not completely understood. It is generally accepted that aging is driven by time accompanied accumulation of molecular and cellular damage. As in diabetic peripheral neuropathy, damage to both large and small fibers can be caused by axonal damage or demyelination.

The limitations of this study should be fully addressed. First of all, the study was conducted in Huashan hospital and T2DM patients recruited were not society based, which could lead to bias since those patients were mostly middle to old age. Secondly, in this cross-sectional study, we used relatively subjective methods to evaluate large nerve dysfunction such as VPT, traditional scoring methods including NSS, NDS and MNSI scores instead of electromyography which has been proved as golden standard in clinical use. Since electromyography is more time consuming and expensive compared to those screening methods, we didn’t perform it in this study. Therefore, further study with more accurate electrophysiological methods could be considered as diagnostic methods in determining the association between age and the incidence of DPN.

As we know, the management of diabetic polyneuropathy includes three main elements: normal blood glucose level, foot care, and treatment of pain according to the guideline of management[6]. The results of this study, demonstrating that advancing age is associated with an increased risk of developing DPN in T2DM patients, emphasize the necessity of an intensified, proactive screening for DPN in elderly patients with T2DM.
